# Adverse Effects of Cholinesterase Inhibitors in Dementia, According to the Pharmacovigilance Databases of the United-States and Canada

**DOI:** 10.1371/journal.pone.0144337

**Published:** 2015-12-07

**Authors:** Thibault B. Ali, Thomas R. Schleret, Brian M. Reilly, Winston Yuchen Chen, Ruben Abagyan

**Affiliations:** Skaggs School of Pharmacy and Pharmaceutical Sciences, University of California San Diego, La Jolla, California, United States of America; University of Bologna & Italian Institute of Technology, ITALY

## Abstract

This survey analyzes two national pharmacovigilance databases in order to determine the major adverse reactions observed with the use of cholinesterase inhibitors in dementia. We conducted a statistical analysis of the Food and Drug Administration Adverse Event Reporting System (FAERS) and the Canada Vigilance Adverse Reaction Database (CVARD) concerning the side effects of cholinesterase inhibitors. The statistics calculated for each adverse event were the frequency and the reporting odds ratios (ROR). A total of 9877 and 2247 reports were extracted from the FAERS and CVARD databases, respectively. A disproportionately higher frequency of reports of death as an adverse event for rivastigmine, compared to the other acetylcholinesterase inhibiting drugs, was observed in both the FAERS (ROR = 3.42; CI_95%_ = 2.94–3.98; P<0.0001) and CVARD (ROR = 3.67; CI_95%_ = 1.92–7.00; P = 0.001) databases. While cholinesterase inhibitors remain to be an important therapeutic tool against Alzheimer’s disease, the disproportionate prevalence of fatal outcomes with rivastigmine compared with alternatives should be taken into consideration.

## Introduction

This study aims to analyze the Food and Drug Administration Adverse Event Reporting System (FAERS) [1] database and the Canada Vigilance Adverse Reaction Database (CVARD) [2], to determine the relative frequencies of adverse events reported during the post-marketing monitoring of cholinesterase inhibitors used in the treatment of dementia.

Dementia has been described as a progressive impairment of cognitive and intellectual faculties caused by disorders affecting the brain [3]. Among the causes of dementia, Alzheimer’s disease (AD) is the most common, representing 60–80% of cases [4]. Over 35 million people suffer from Dementia worldwide, with 7.7 million new cases diagnosed each year, [5] and due to the global aging of populations, it has been estimated that the number of people affected by dementia will reach around 115 million by 2050 [6][7]. The rates of increase are not uniform from one country to another and they are projected to increase by 300% in developing countries while 100% in developed countries, making dementia and AD an international challenge for the future [8][9].

The current treatment options for AD are focused on the alleviation of symptoms and extending patient autonomy. Among the treatments, the cholinesterase inhibitor class are used for mild to moderate Alzheimer’s disease and related dementia. Several controlled clinical trials based on a specially selected population have tested these three drugs and showed them to be of comparable efficacy and safety [10][11][12]. However, due to the limited population tested in clinical trials, some rare adverse reactions may not surface until an extensive body of post-marketing data is accumulated. Since the cholinesterase inhibitors were introduced for treatment of Alzheimer’s disease, thousands of adverse event reports have been submitted to the FAERS and CVARD databases.

The post-marketing monitoring has proved its usefulness in revealing serious adverse effects missed in clinical trials and many drugs have been either removed from the market or have seen warnings added to their labels due to post marketing reports of previously unknown or insufficiently quantified serious adverse events (CDER 2005 Report to The Nation, 2005). Some examples include Tetrazepam [13], a benzodiazepine derivative with anxiolytic and muscle relaxant properties that has been removed from the European market in July 2013 because of rare but serious life-threatening skin reactions. However, the duration of time that drugs are on the market prior to their removal varied widely. For example, temafloxacin (Omniflox) was removed after 4 months, whereas propoxyphene (Darvocet) was not removed until 55 years after it was approved. Discrepancies like these indicate that a timely analysis of post-marketing data is critical for drug safety.

In 2010, Novartis and the FDA highlighted the fact that Healthcare Professionals and Caregivers had to be careful about the proper use and application of Exelon^®^ Patch (rivastigmine transdermal system). Indeed, due to several errors of medication with the patch form, serious adverse events that have required hospitalization and sometimes led to death have been reported [14].

To better understand these observations, we first focused our work on the adverse events of cholinesterase inhibitors reported to the FDA Adverse Event Reporting System (FEARS) database using a searching tool that significantly speeds and helps organize the data (Early Drug Alert^™^, developed by Abagyan Lab at UCSD) [15]. Secondly, reports from the Canada Vigilance Adverse Reaction Database were analyzed with a manual searching method, in order to compare the results from the FDA FEARS with another large-scale patient population.

## Methods

This study is a retrospective data analysis of adverse event reports based on the FDA Adverse Event Reporting System Database (FAERS) and the Canada Vigilance Adverse Reaction Database (CVARD). The disease classification used for both US and Canadian databases is based on the MedDRA classification. Only reports with drug codes of “Primary Suspect” or “Secondary Suspect” from the FAERS database were included in our analysis, all other drug codes were ignored.

### FDA adverse event reporting system

FAERS, created in 2004, is a database containing adverse event and medication error reports sent to the FDA. The reports of adverse event and medication error are voluntarily reported by healthcare professionals and consumers to the FDA, either directly via MedWatch or indirectly via drug manufacturers [16]. Clinical reviewers in the Center for Drug Evaluation and Research (CDER) and the Center for Biologics Evaluation and Research (CBER) use this database to monitor the safety of drugs after the FDA approval. However, FAERS is a public database and the FDA provides data files upon request [17]. The data analyzed in this study are the reports from the 2004 Q1 data to the 2012 Q3 data for the 3 FDA approved acetylcholinesterase inhibitors: rivastigmine, donepezil and galantamine. The data from FAERS was organized into a rational searchable interface by the Abagyan Lab at the UCSD Skaggs School of Pharmacy, and subsequently named “Early Drug Alert” [15]. This resource presents a special analysis of the primary data in which the adverse effects and indications are grouped into categories; and baseline frequencies of adverse effects for comparison between different drugs with the same indication are calculated together with their variations.

### Canada Vigilance Adverse Reaction Database

The Canadian Vigilance Adverse reaction database, created in 1965, contains reports submitted by health professionals, consumers, manufacturers and distributors. Seven Canadian Vigilance Regional offices collect reports and the Canada Vigilance Program supervises the whole database. The Canada Vigilance Program uses this database to monitor the safety of drugs once they are marketed. The data are accessible to public and can be extracted from their online platform [18][19]. In this study, we extracted all the reports available in CVARD for rivastigmine, donepezil and galantamine occurring between January 1^st^ 1990 to January 1st 2014.

### Statistical Analysis

Both FAERS and CVARD data were analyzed the same way. The statistics calculated were the frequencies of the most highly reported adverse effects of rivastigmine, donepezil, galantamine and the acetylcholinesterase inhibitor (AChEI) class (which includes reports from all three AChEI’s). The FAERS database reports death, sudden death, sudden cardiac death, cardiac death, brain death, accidental death and apparent death as different adverse events, while the CVARD indicates the term ‘death’ for any fatal outcome. In order to compare the two different databases, we regrouped all the fatal outcomes from FAERS database into a single term, “death”. The reports citing drug combinations were excluded to simplify the statistical analysis of primary suspects and secondary suspect.

The relative odds of a particular AChEI having a particular adverse event report in comparison to its drug class as a whole were expressed as Reporting Odds Ratios (ROR) with 95% confidence intervals (CI) and were calculated and using MedCalc^®^ software (version 15.2) [20][21]. Among four widely used data mining algorithms, ROR showed the best performance for pharmacovigilance surveys [22][23]. Indeed, ROR is generally used to evaluate disproportion in reporting AEs for a drug in comparison with all other drugs and it has been very useful for many pharmacovigilance studies [24]. It allows an estimation of relative signal, giving a general idea of how much more frequently a particular AE is associated with a drug in comparison to other comparable drugs for the same indication.

It is important to note that the FAERS and CVARD do not allow for the derivation of the absolute adverse effect frequencies because the reporting is voluntary and the total number of prescriptions is not known. In the analysis below, the baselines used for the following rates and frequencies calculated correspond to the total number of adverse effects reported. This is why the rates and frequencies calculated are considered as relative because it does not reflect the frequency or rates of a specific adverse effect for the acetylcholinesterase inhibitor users, but only for users who made a report of an adverse event.

## Results

### FAERS Database

FAERS database included a total of 3661205 reports from January 2004 to January 2012. It contains 9877 reports of side effects for rivastigmine, donepezil and galantamine. Rivastigmine had the highest number of reports with 5918 reports, while donepezil and galantamine had respectively 2221 and 1738 reports (Table 1).

**Table 1:** Top adverse effects for cholinesterase inhibitors in FAERS database.

Top Adverse Effects	Drug specific reports	AChEI class reports	Specific drug frequency (%)	AChEI class frequency (%)
**rivastigmine– 5918 total reports**				
**Death**	**995**	**1216**	**16.81**	**12.31**
Vomiting	472	690	7.98	6.99
Fall	417	638	7.05	6.46
Nausea	374	557	6.32	5.64
Confusional State	361	475	6.10	4.81
Dizziness	256	381	4.33	3.86
Pneumonia	253	373	4.28	3.78
Diarrhoea	245	375	4.14	3.80
Hallucination	224	305	3.79	3.09
Malaise	219	304	3.70	3.08
**donepezil– 2221 total reports**				
Bradycardia	178	338	8.01	3.42
Fall	139	638	6.26	6.46
Vomiting	122	690	5.49	6.99
Syncope	118	343	5.31	3.47
Convulsion	99	240	4.46	2.43
Nausea	99	557	4.46	5.64
**Death**	**95**	**1216**	**4.28**	**12.31**
Electrocardiogram QT prolonged	94	130	4.23	1.32
**Rhabdomyolysis**	**94**	**114**	**4.23**	**1.15**
Drug Interaction	86	183	3.87	1.85
**galantamine– 1738 total reports**				
**Death**	**126**	**1216**	**7.25**	**12.31**
Vomiting	96	690	5.52	6.99
Nausea	84	557	4.83	5.64
Fall	82	638	4.72	6.46
Medication Error	71	102	4.09	1.03
Decreased Appetite	64	304	3.68	3.08
Loss of consciousness	61	248	3.51	2.51
Dizziness	59	381	3.39	3.86
Bradycardia	57	338	3.28	3.42
Confusional State	56	475	3.22	4.81

**Fig 1:**
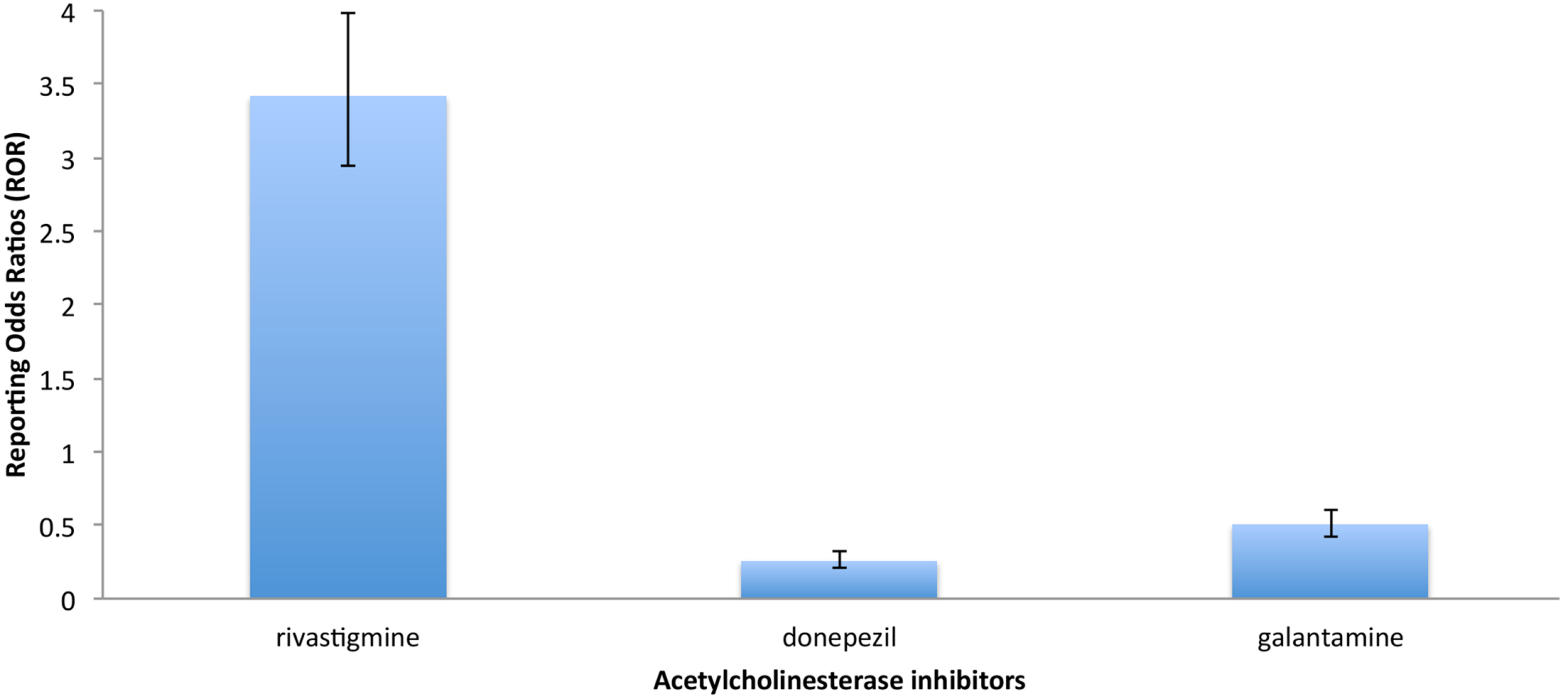
Rates of death for specific ChEI in the FAERS database. Reporting odds ratios comparing the rates of death for specific ChEI to the rates for the general ChEI drug class.

The association between the use of AChEI and serious side effects is shown in Table 2. Reporting odds ratios of Table 2 compare the rates of serious adverse events in specific drug to the rates for the general AChEI drug class. Overall, death was more often reported in patients using rivastigmine (ROR = 3.42; CI_95%_ = 2.94–3.98; P<0.0001) and rhabdomylosis was more often reported in patients using donepezil (ROR = 11; CI_95%_ = 10.39–27.41; P<0.0001) in the FAERS database. Fig 1 shows the reporting odds ratios (ROR) comparing the rates of death in specific AChEI to the rates for the general AChEI drug class.

**Table 2:** FAERS database analysis of reported serious adverse effect.

Adverse Effect	Cases	ROR (95% CI)	*p* value
**rivastigmine**			
**Death**	**995**	**3.42 (2.94–3.98)**	**<0.0001**
Fall	417	1.28 (1.08–1.52)	0.0038
Pneumonia	253	1.43 (1.15–1.78)	0.0016
Syncope	175	0.69 (0.55–0.85)	0.0007
Loss of Consciousness	128	0.71 (0.55–0.91)	0.0071
Convulsion	106	0.52 (0.40–0.67)	<0.0001
**Rhabdomyolysis**	**11**	**0.07 (0.04–0.13)**	**<0.0001**
**donepezil**			
**Death**	**95**	**0.26 (0.21–0.32)**	**<0.0001**
Fall	139	0.96 (0.79–1.16)	0.6616
Pneumonia	70	0.79 (0.61–1.03)	0.0800
Syncope	118	1.87 (1.49–2.34)	<0.0001
Loss of Consciousness	59	1.08 (0.80–1.45)	0.6185
Convulsion	99	2.49 (1.91–3.23)	<0.0001
**Rhabdomyolysis**	**94**	**16.87 (10.39–27.41)**	**<0.0001**
**galantamine**			
**Death**	**126**	**0.51 (0.42–0.61)**	**<0.0001**
Fall	82	0.68 (0.53–0.86)	0.0012
Pneumonia	50	0.72 (0.53–0.97)	0.0309
Syncope	50	0.79 (0.59–1.08)	0.1358
Loss of Consciousness	61	1.55 (1.15–2.08)	0.0036
Convulsion	35	0.80 (0.55–1.14)	0.2156
**Rhabdomyolysis**	**9**	**0.40 (0.20–0.79)**	**0.0082**

### Canada Vigilance Adverse Reaction Database

In January 2014, The Canadian Vigilance Adverse reaction database included a total of 2247 reports for the AChEI drug class: 1396 reports for rivastigmine, 602 reports for donepezil and 249 reports for galantamine.

**Fig 2:**
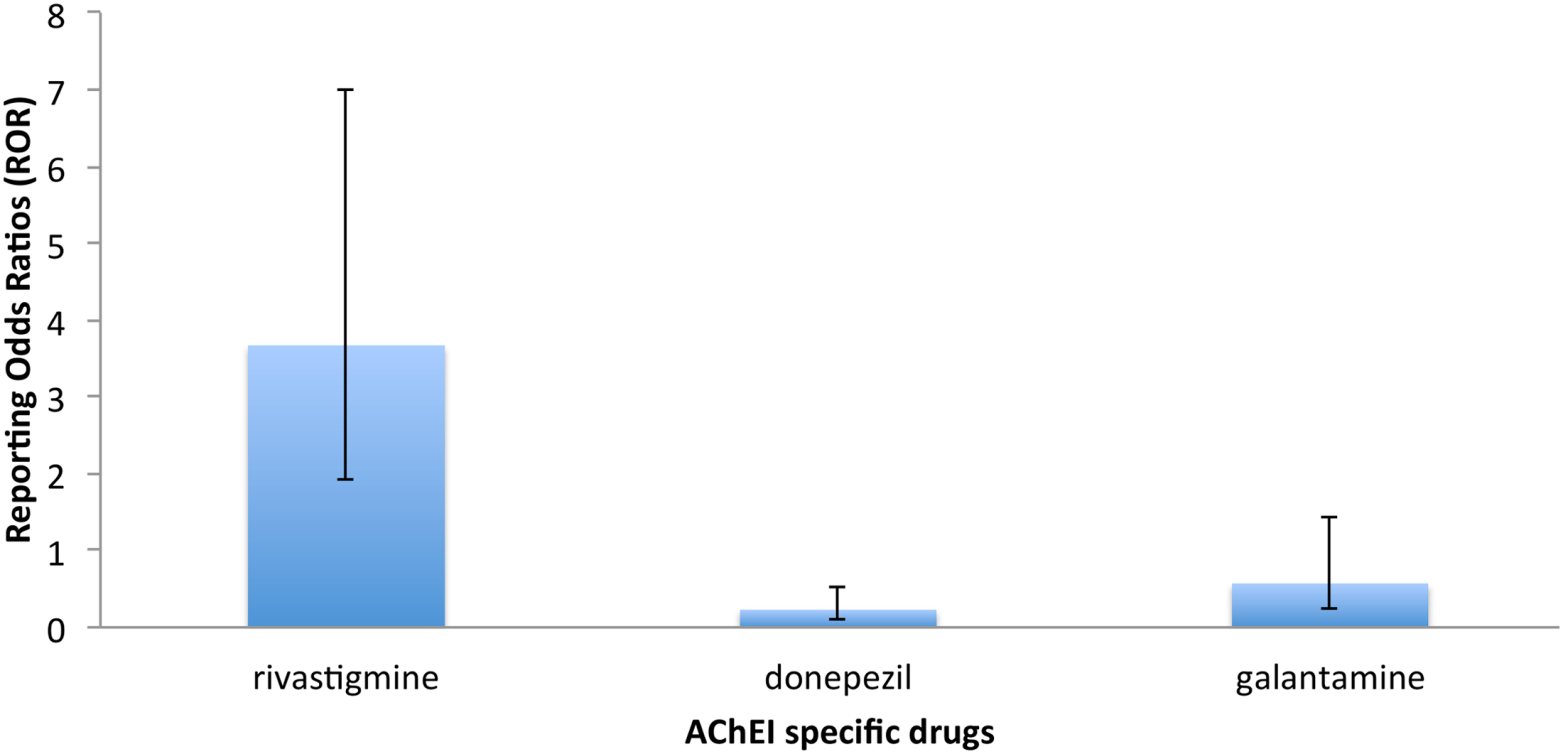
Rates of death for specific ChEI in the Canada Vigilance Adverse Reaction database. Reporting odds ratios comparing the rates of death for specific ChEI to the rates for the general ChEI drug class.

ROR in Table 3 compares the rates of death in specific AChEI to the rates for the general AChEI drug class in the Canada Vigilance Adverse Reaction database. Death was more often reported in patients using rivastigmine (ROR = 3.67; CI_95%_ = 1.92–7.00; P = 0.001) than donepezil or galantamine. Fig 2 shows a histogram representation of the reporting odds ratios comparing the rates of death in specific AChEI to the rates for the general AChEI drug class.

**Table 3:** Analysis of death reports in the Canada Vigilance Adverse Reaction database.

Drug name	Cases	ROR Death (95% CI)	P-value
Rivastigmine	64	3.67 (1.92–7.00)	0.0001
Donepezil	6	0.23 (0.10–0.54)	0.0006
Galantamine	5	0.57 (0.23–1.43)	0.2317

## Discussion

These results are subject to an underreporting bias limitation. Indeed, neither the total number of prescription of cholinesterase inhibitors nor the relative numbers of prescriptions for rivastigmine, donepezil and galantamine within the cholinesterase inhibitors group are known because these data are not made available to the public by FAERS or CVARD. Moreover, all reports used from the FAERS and CVARD are voluntary and doesn’t represent the totality of adverse events induced by cholinesterase inhibitors. Thus, the results obtained are considered relative because data used are not based on the total of cholinesterase inhibitor prescriptions but on the total number of voluntary adverse events reports.

According to the results from the FAERS database Reporting Odds Ratios (Table 2), *p* values for death reports indicate significant results for all cholinesterase inhibitors (*p* value < 0.0001). Regarding the relative odds ratios, death was more often reported in patients using rivastigmine (ROR = 3.42; CI_95%_ = 2.94–3.98; P<0.0001) than for patients using donepezil (ROR = 0.26; CI_95%_ = 0.21–0.32; P<0.0001) and galantamine (ROR = 0.51; CI_95%_ = 0.42–0.61; P<0.0001). Furthermore, the Canadian Vigilance Adverse Reaction Database confirms this observation with patients using rivastigmine (ROR = 3.67; CI_95%_ = 1.92–7.00; P = 0.0001) (Table 3).

Although we need to take into account that the increased death reports with rivastigmine might be influenced by the fact that rivastigmine is more often prescribed for patients with an advanced stage of Alzheimer’s Disease, other hypotheses can be made. In order to further investigate the underlying cause of our findings, we first determined what sets rivastigmine apart from the other AChEIs. The most glaring difference between rivastigmine and the other AChEIs is that rivastigmine is the only drug among the cholinesterase inhibitors to have the transdermal patch administration form. [25]. It has been reported that improper use of the transdermal administration form may lead to higher than recommended administered doses.[26]. As explained in Novartis’ warning letter from the FDA in 2007 [14], a majority of medication errors have involved not removing the old patch prior to applying a new one, leading to the application of multiple patches at one time. Indeed, a 2012 report from Henrik Lövborg, Anna K Jönsson and Staffan Hägg [26] illustrates a patient’s fatal outcome involving a rivastigmine overdose due to a medication error with the patch form. The patient described in the study was an 87-year-old male with dementia. He developed nausea, vomiting and renal failure with disturbed electrolytes resulting in death. These events occurred after six rivastigmine patches had concomitantly and erroneously been applied by health care personnel on two consecutive days.

Another difference between rivastigmine compared with the other cholinesterase inhibitors relates to the molecular mechanism of action of these drugs. The acetylcholinesterase inhibitors are thought to act via the inhibition of the catabolic acetylcholinesterase enzyme, leading to increased acetylcholine availability at the synaptic cleft of cholinergic neurons. [27]. Although the mechanism of action of these three drugs is common, there are pharmacological differences that must be taken into account because of the possible repercussions on the tolerance. Both donepezil and galantamine are reversible inhibitors of acetylcholinesterase, with a non-covalent binding to the enzyme [28][29]. Rivastigmine, on the other hand, has been classified as an intermediate-acting agent (pseudo-irreversible) [30]. However, a study on the kinetic and structural interaction of rivastigmine with the acetylcholinesterase enzyme has shown that rivastigmine led to an extremely low reactivation of the acetylcholinesterase enzyme [31]. The very tight bond observed between rivastigmine and the acetylcholinesterase enzyme active site appeared to be almost irreversible and could potentially lead to an unexpected increase in the duration of action.

Regarding other adverse effects uncovered during this study, donepezil was predominantly associated with muscle related adverse effects. The reporting odds ratios from FAERS(Table 2) indicate that donepezil is highly associated with reports of rhabdomyolysis in comparison to other AChEIs (ROR = 16.87; CI_95%_ = 10.39–27.41; P<0.0001). In support of our findings, a recent case report concluded that an acute renal failure secondary to rhabdomyolysis was probably induced by donepezil [32]. In this case an 84-year-old male patient with Alzheimer’s disease who had been taking donepezil 5mg daily for two months was admitted to the emergency department for apathy, loss of cooperation and decreased muscle strength. Initially, the patient’s kidney function indicated an acute renal failure, and donepezil was discontinued. After discontinuation of donepezil, the patient’s renal function improved gradually until becoming normal again after 12 days of care. According to author suggestions, the acute renal failure was not a direct effect but may have been mediated by the necrotic myolytic effect of donepezil.

The results obtained from both FAERS and CVARD were statistically significant and consistent across both databases, in addition to being supported by case reports from clinicians. It would have been even more reassuring to consider other national sources of pharmacovigilance, however the differences in reporting policies, data accessibility, data access cost, data formats and the absence of uniform vocabulary made further analysis difficult.

In conclusion, acetylcholinesterase inhibitors are efficient symptomatic treatments against dementia, however we need to consider the risk profiles of each of these drugs so that the safest drug can be chosen for each patient. The data from this study indicate an association between rivastigmine and rare events of fatal outcomes, as well as donepezil and rhabdomyolysis. We hope that these findings will help healthcare professionals to make more informed decisions for their dementia patients.
